# Pair cross-correlation analysis for assessing protein co-localization

**DOI:** 10.1016/j.bpj.2025.03.002

**Published:** 2025-03-12

**Authors:** Pintu Patra, Cecilia P. Sanchez, Michael Lanzer, Ulrich S. Schwarz

**Affiliations:** 1 Institute for Theoretical Physics, Heidelberg University, Heidelberg, Germany; 2 BioQuant, Heidelberg University, Heidelberg, Germany; 3 Department of Physics, Indian Institute of Technology Kharagpur, Kharagpur, India; 4 Department of Infectious Diseases, Parasitology, Universitätsklinikum Heidelberg, Heidelberg, Germany

## Abstract

Measuring co-localization of different types of molecules is essential to understand molecular organization in biological systems. The pair cross-correlation (PCC) function computed from two-color microscopy images provides a measure of co-localization between differently labeled molecules. Here, we compute a theoretical expression for the PCC function between two molecules using two-dimensional Gaussian distributions as the effective point-spread functions for single molecules. Through our analytical calculations, we provide a quantitative description of PCC in the case of multiple signal pairs. By fitting our analytical solutions to simulated images, we can estimate both small and large separation distances. We then apply this method to malaria-infected red blood cells (RBCs) imaged by stimulated emission depletion (STED) microscopy. We cross-correlate the signal for the knob-associated histidine-rich protein, which the parasite uses to remodel the spectrin-actin network of RBCs, with different signals from the RBCs and find that its average separation from the ankyrin junctions increases from 40 nm to 120 nm during the 48 h of the infectious cycle.

## Significance

With the advent of super-resolution microscopy, it has become possible to extract information on the nanometer-scale organization of biomolecules in cells from imaging data. A natural way to quantify the co-localization of two types of molecules is the pair cross-correlation (PCC) function. By developing a theoretical framework for PCC based on the assumption of two-dimensional Gaussian point-spread functions, we provide a better understanding of PCC profiles and describe a method to estimate separation distances between molecules through image analysis. Our approach is first validated through simulated images and then applied to malaria-infected red blood cells, where we quantify the re-localization of a central protein used by the parasite. This case study demonstrates that PCC is a useful method to extract quantitative molecular information from super-resolution images.

## Introduction

Dynamic organization of molecules in cells is key to virtually all cellular functions (1). Most complex cellular functions, such as adhesion, migration, division, and sensing, requires efficient organization of the involved molecules in space and time. Measuring molecular changes during these biological processes is critical for their understanding and for biomedical applications. During the last two decades, super-resolution microscopy has revolutionized our abilities to image on ever-decreasing scales (2,3). Dual-color super-resolution microscopy enables the measurement of the distance between different types of molecules with high precision (4,5). With current methods, one can resolve distances down to a few nanometers in specialized situations; for more general applications, the current resolution is tens of nanometers (6).

To extract meaningful conclusions and parameters related to biological systems or processes of interest, one requires methods to analyze the acquired microscopy data. Several methods that determine the spatial correlation between two differently labeled fluorophores are localization based (7,8). Recently, an image-based cross-correlation analysis method has been used to show the extent of spatial co-clustering of two proteins to each other (9,10). Mathematically both approaches, i.e., particle localization-based distance distributions and pixel-based correlation function for super-resolution microscopy data, are equivalent and their usage depends on context (9). Here we focus on the second method, which computes a pair cross-correlation (PCC) function to quantify spatial organization from dual-color images. The PCC function for an image containing multiple pairs of differently labeled molecules measures the probability of finding one molecule at a distance *r* from another molecule compared to a sample with a random distribution of the two molecules (8).

The PCC method provides a metric to describe changes in the spatial organization of molecules in different samples (11) and in different physiological conditions (7,8). For example, a high degree of PCC at a given spatial distance *r* indicates the presence of spatial arrangement or interaction between molecules, whereas the absence of such correlation suggests random spatial arrangement and nonspecific interactions (7). The use of PCC to quantify temporal changes in spatial organization during biological processes, development of disease, or transport of molecules has so far not been explored much. Our previous work has made an attempt in this direction by using the PCC-based analysis to show the dynamic re-localization of the knob-associated histidine-rich protein (KAHRP), a central parasite protein that binds to the red blood cell (RBC) cytoskeleton, during malaria infection (10,12). KAHRP is a central building block of the thousands of adhesive knobs that are induced by the parasite on the RBC surface to increase residency time in the vasculature and to avoid clearance by the spleen (13,14).

The RBC cytoskeleton is composed of a quasi-hexagonal network formed by spectrin tetramers that are connected by junctional complexes with actin protofilaments. The spectrin-actin network is connected to the plasma membrane both at these junctional complexes and at the ankyrin bridges at the midpoints of the spectrin tetramers. We studied the spatial localization of several proteins (including KAHRP, actin, ankyrin, and others) during the remodeling of the RBC cytoskeleton in different states of malaria infection using stimulated emission depletion (STED) microscopy (10,12). Specifically, we found that, at the initial ring stage (1–24 h post invasion), KAHRP co-localizes both with the ankyrin bridges and the junctional complexes, and its PCC with ankyrin shows a maximum at zero distance. At the end of the ring stage and the beginning of the trophozoite stage (24–36 h post invasion), it re-localizes to the actin junctional complexes, indicated by the presence of a peak at finite separation in the PCC profile between ankyrin and KAHRP. This suggests that knobs are formed predominantly at junctional complexes, in agreement with the observation that the parasite dissolves the actin protofilaments (15,16). Although the peak at a finite separation provides an estimate of separation distance, the peak at origin for co-localizing signals provides only a qualitative measure of proximity between the signals. Hence, we concluded that KAHRP localization with respect to ankyrin is dynamic during progression of the infection. However, we could not estimate the changes in actual separation or other signal parameters from the experimental images. Only for larger separations, such as molecules at different positions along the relatively long spectrin filaments, did PCC profiles show a clear peak, and centroid-to-centroid distances could be measured, as reported in our earlier work on changes in the RBC cytoskeleton during the time course of a malaria infection (10). In general, in all previous studies, the PCC profile provided more qualitative changes between different conditions rather than quantitative measures (7,8,9). Furthermore, the qualitative understanding of changes in the PCC profile lacks insight into the influence of image features, such as shape, size, and density of signals, which can potentially modulate the PCC profile for a given image. This limitation stems from the reliance on a limited number of images under specific conditions rather than diverse samples with wide variability.

Here, we address these issues by theoretically connecting the definition of PCC for two molecules using two-dimensional (2D) Gaussian distributions as the effective point-spread functions for single molecules to the PCC of multiple molecule pairs in a given image. We use this approach to dissect the effect of different relevant image and signal parameters on the PCC profile through several examples. We further describe a way to extract the effective or average separation distances between molecule pairs from the PCC profile. To show the effectiveness of our analysis, we extract the separation distance and signal size for KAHRP and ankyrin during the infectious cycle (10). In summary, we utilize both experimental and simulated images to understand the relationship between the PCC profile and various biologically relevant molecular parameters, aiming to improve the quantification of spatial organization in two-color images.

## Materials and methods

### In vitro culture of *Plasmodium falciparum*

Malaria is caused by the infectious agents from the genus *Plasmodium*, with *P. falciparum* being the most deadly species. The *P. falciparum* clonal line FCR3 was cultured according to the protocol established by Trager and Jensen (17). Fresh A+ erythrocytes were resuspended in RPMI 1640 medium supplemented with 25 mM HEPES, 2 mM L-glutamine, 0.2 mM hypoxanthine, 5% (v/v) human serum, 5% (v/v) GlutaMAX, 20 *μ*g/mL gentamycin (Thermo Fisher Scientific), and 5% human serum. The cultures were maintained at a hematocrit of 4%, parasitemia levels below 5%, and incubated at 37°C under a gas mixture of 5% ${O\;}_{2}$, 3% ${\text{CO}\;}_{2}$, and 92% ${N\;}_{2}$, with a relative humidity of 96%.

### Preparation of exposed membranes

Exposed membranes were prepared following the protocol described by Sanchez et al. (10). Briefly, glass-bottom culture dishes (MatTek Corporation) were treated with 2% 3-aminopropyl triethoxysilane in 95% ethanol for 10 min, followed by rinsing with 95% ethanol. The dishes were then incubated at 100°C for 15 min before being treated with 1 mM bis(sulfosuccinimidyl) suberate in phosphate-buffered saline (PBS) at room temperature for 30 min (18). After washing with PBS, the dishes were treated with 0.1 mg/mL phytohemagglutinin E in PBS, as previously described (18). The dishes were again washed with PBS, blocked with 0.1 M glycine for 15 min, and washed once more with PBS before being stored at 4°C until further use. Magnetically enriched *P. falciparum*-infected erythrocytes were immobilized on the functionalized dishes followed by washes with hypotonic phosphate buffer (10 mM sodium phosphate, pH 8, 10 mM NaCl) and water (19). The exposed membranes were fixed with 4% paraformaldehyde in PBS for 15 min, washed with PBS, and blocked in PBS containing 3% bovine serum albumin (BSA). Membranes were incubated with primary antibodies overnight at 4°C and with secondary antibodies for 40 min at room temperature. All incubations and washes were performed in PBS with 3% BSA. The following primary and secondary antibodies were used: mouse monoclonal KAHRP antibody 18.2 (0.8 *μ*g/mL, The European Malaria Reagent Repository); rabbit KAHRP288-302 antiserum (1:500, custom-made by Eurogentec); mouse monoclonal ankyrin-1 antibody H-4 (1:200, Santa Cruz Biotechnology); Star 580 goat anti-mouse antibody (1:200, Abberior); and Star Red goat anti-rabbit antibody (1:200, Abberior).

### STED microscopy

Super-resolution images were acquired using an STED/RESOLFT microscope (Abberior Instruments, Germany) equipped with 488 nm, 594 nm, and 640 nm excitation lasers, along with a 775 nm STED laser. An Olympus microscope with a 100× oil immersion objective (UPLSAPO 1.4 NA oil, 0.13 mm WD) was used. The STED laser power was set to 40%. Fluorescence emitted from Abberior Star 580-conjugated secondary antibodies was detected in the 594 nm excitation channel (green images), whereas fluorescence from Abberior Star Red-conjugated secondary antibodies was detected in the 640 nm excitation channel (magenta images). The pixel size was set to 15 nm, with a pixel dwell time of 10 *μ*s. Deconvolution of 2D-STED images was performed using Imspector software (Abberior Instruments), utilizing the Richardson-Lucy algorithm with default settings and a regularization parameter of 1 × 10^−10^.

## Results

### PCC between two Gaussian signals

To provide a theoretical understanding of PCC, we first calculate analytically the PCC profile for two Gaussian signals. The steps for computing pair cross-calculation from images and localization points are detailed in previous studies (9,20). In summary, the PCC function between a pair of images is computed by first calculating the pair-distance distribution (PDD). The PDD for localization-based data is a histogram of pair distances of differently labeled molecule pairs (8,9,20). For image-based data, it is defined as the sum of pixel intensity products of the two images for fixed radial separation (obtained by different possible translational shifts in x and y directions between the images for a fixed euclidean distance between them) normalized by the product of the total intensities of the individual images (9). The PCC profile is calculated by binning the resultant distribution using annular bins (details described in the appendix). Here we drive the PCC profile for two Gaussian signals with standard deviations $\sigma_{1}$ and $\sigma_{2}$, respectively, and separated by a distance *μ*. PDD between two Gaussian signals has been previously calculated (4,21) and recapitulated here for completeness. For two Gaussian signals (with distribution $p\left({\vec{r}}_{i},{\vec{\mu }}_{i},\sigma_{i}\right)$) with a peak at ${\vec{\mu }}_{1}$ and ${\vec{\mu }}_{2}$, the probability of finding a pair at a separation distance, $\vec{r}={\vec{r}}_{1}-{\vec{r}}_{2}$, is a Gaussian distribution with mean $\mu =\left|\vec{\mu }\left|=\right|{\vec{\mu }}_{1}-{\vec{\mu }}_{2}\right|$ and variance $\sigma^{2}=\sigma_{1}^{2}+\sigma_{2}^{2}$ and has the following form:

(1) $$P_{12}\left(r,\phi \right)=\frac{1}{2\pi \sigma^{2}}\;\exp\;\left(-\frac{{\left(\vec{r}-\vec{\mu }\right)}^{2}}{2\sigma^{2}}\right)=\frac{1}{2\pi \sigma^{2}}\;\exp\;\left(-\frac{\left(r^{2}+\mu^{2}-2r\;\mu \;\cos\;\phi \right)}{2\sigma^{2}}\right)\;\text{.}$$

Integrating over all possible angles (*ϕ*) in two dimensions (0 to 2π), all pairs with fixed separation *r* can be sampled. Hence, we can obtain the probability for observing a given separation distance in 2D, the PDD, as

(2) $$P\left(r\right)=\int_{0}^{2\pi }rd\phi P_{12}\left(r\right)=\frac{r}{\sigma^{2}}\;\exp\;\left(-\frac{\mu^{2}+r^{2}}{2\sigma^{2}}\right)I_{0}\left(\frac{r\mu }{\sigma^{2}}\right)$$

where $I_{0}\left(x\right)=\left(1/\left(2\pi \right)\right)\int_{0}^{2\pi }d\phi \;\exp\;\left(x\;\cos\;\phi \right)$ is the modified Bessel function of integer order zero.

In the next step, the above PDD is binned into radial bins. The binned distribution for infinitesimally small bin-width Δr, can be written as

(3) $$P_{bin}\left(r\right)=\Delta r\;\frac{r}{\sigma^{2}}\;\exp\;\left(-\frac{\mu^{2}+r^{2}}{2\sigma^{2}}\right)I_{0}\left(\frac{r\mu }{\sigma^{2}}\right)\;\text{.}$$

Finally, the PCC is computed by normalizing the binned distribution with the relative area of the annular bins as follows:

(4) $$C\left(r\right)=\frac{P_{bin}\left(r\right)}{A_{bin}/A_{image}}=P_{bin}\left(r\right)\times \frac{A_{image}}{\pi \Delta r\left(2r+\Delta r\right)}=\frac{A_{image}}{2\pi \sigma^{2}}\;\exp\;\left(-\frac{\mu^{2}+r^{2}}{2\sigma^{2}}\right)I_{0}\left(\frac{r\mu }{\sigma^{2}}\right)$$

where in the last expression we have set Δr=0, assuming infinitesimal small bin size.

**Figure 1:**
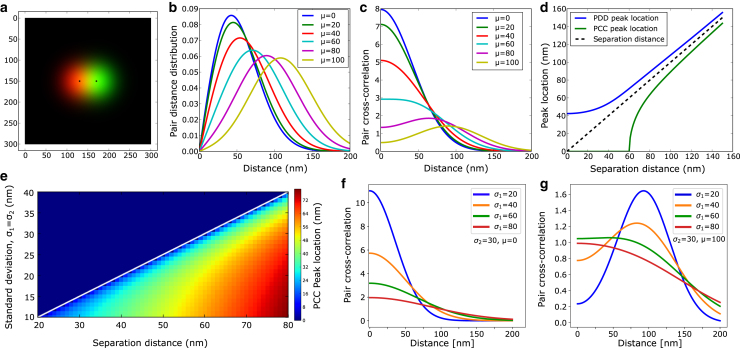
Differences between a PDD and PCC distribution. (*a*) A pair of fluorescent signals (*red and green*) separated by 40nm. The standard deviation for the red and green signals is set to 25nm and 30nm, respectively. (*b*) PDD for two Gaussian signals separated by different distances from 0 to 100nm. The peak of the PDD at a smaller separation distance shows more minor shifts and appears to be saturated for a distance of less than 20nm. (*c*) PCC for two Gaussian signals at different distances. For zero separation, the PCC value at the origin is the highest. It decreases progressively as the separation distance increases. For separation distance >60nm, the PCC shows a peak at an intermediate distance. The peak distance is close to the actual separation between the signals. (*d*) The location of peaks in PDD and PCC profiles is compared with the exact separation distance. The peak location of PDD profiles is higher than the actual separation and saturates at a finite value at smaller separation distances. The peak locations of PCC profiles are lower than the actual separation at large separation distances and go to zero for smaller separation distances. (*e*) The phase diagram separates the regime with and without a peak in the PCC profile. The heatmap shows the value of the PCC peak distance. The solid line separating the two regimes is given by $\mu \approx \sqrt{2}\sigma$. (*f*) Effect of change in standard deviation of Gaussian signals on PCC for zero separation distance. (*g*) Same as in (*f*) with separation distance μ=100nm.

The above analytical expression for PCC provides a simple way to interpret changes in the PCC profile, e.g., changes in the location of the peak in the PCC profile under different parameters and conditions. The above expression shows that the PCC profile C(r) is determined by the variation of two functions, a decreasing exponential function and another increasing function (starting from 1 as $I_{0}\left(0\right)=1$). The separation distance *μ* and *σ* determines if C(r) is purely decreasing or displays a peak. For μ→0, $I_{0}\approx 1$ (remains close to 1) and the PCC profile is a decreasing exponential whose decay is determined by *σ*. For large μ≫0, the exponential function decreases modestly for r<μ and cannot diminish the increasing effect of $I_{0}$, which causes C(r) to increase at small *r* values. However, for large *r* values, the exponential function dominates over $I_{0}$ function and causes the C(r) value to decrease again. Therefore, one observes a peak in C(r) under these conditions. This transition from a peak at zero to a peak at a finite distance in the PCC profile is depicted in Fig. 1 for a pair of simulated Gaussian signal (Fig. 1 *a*). Fig. 1 shows PDD and PCC profile for two Gaussian signals separated by different separation distance. The PDD profile shows a peak at all separation distances. The location of the peak corresponds to the actual separation distance for large separations between the signals. However, for small separations, the peak location saturates and the changes in PDD are insignificant, which makes this distribution (Eq. 2) unreliable for the estimation of separation distance (5). In contrast to PDD, the PCC profile shows progressive changes, its value at the origin decreases as the separation distance increases, and a peak emerges for large separation distances (shown in Fig. 1 *c*). Although PDD profile displays a maximum close (but slightly higher) to the real separation between the two signals, the PCC maximum is slightly lower than actual separation ( $r_{peak}\approx \mu \left(1-\sigma^{2}/2\mu^{2}\right)$ in the limit μ≫σ; Fig. 1 *d*). Therefore the PDD peak saturates to a finite value and the PCC peak disappears as the separation distance decreases.

The presence and absence of a peak in the PCC profile as a function of *σ* (signal standard deviation) are shown in Fig. 1 *e* (upper and lower regions, respectively). The boundary that separates these regions is given by $\mu \approx \sqrt{2}\sigma$ (white line in Fig. 1 *e*). The color code shows the value of the PCC peak distance. The transition from a peak at the origin to a peak at a finite distance in the PCC profile can occur not only through changes in separation distance but also through changes in signal size, for example, due to clustering of molecules during some biological processes. The effect of changes in signal standard deviation is shown in Fig. 1 *f* for zero and nonzero (100nm) separation between the signal pairs. For zero separation, a peak at the origin always exists because the condition $\sqrt{2}\sigma >0$ holds for all *σ* values (clearly seen in Eq. 4 for μ=0). For finite separation, the transition occurs when $\sigma >\mu /\sqrt{2}$, as shown in Fig. 1 *g*.

**Figure 2:**
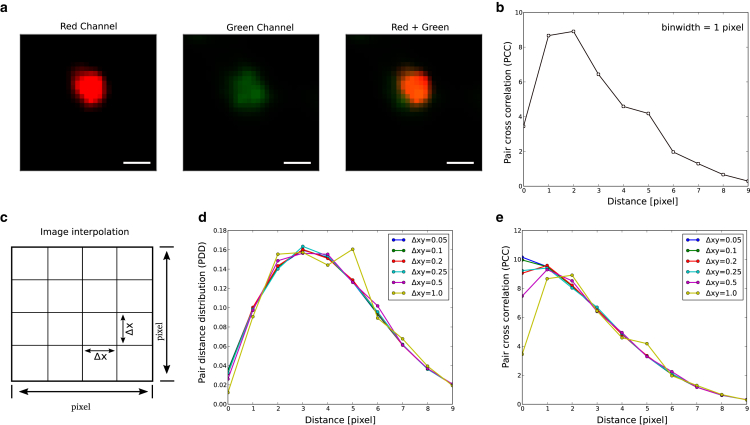
Image interpolation improves the PCC profile. (*a*) A pair of fluorescent signals (red and green channels in the left and center channels, respectively) with overlapping distribution (shown in the right panel). Scale bar, 10 pixels. (*b*) The PCC profile of this image for different distances. (*c*) Image intensity at four corners of a pixel is interpolated to smaller grids (i.e., the pixels of the new image). (*d*) Calculated PDD profiles of the interpolated images at different new pixel widths Δ xy. (*e*) PCC profile converges for images with lower pixel dimensions.

In this work, we focus on the image-based computation of the PCC profile. The pixel-based definition of PDD and PCC and its relation to localization-based data have been defined in our previous work (10) and given in the supporting material for completeness. For image-based data, the PDD and PCC distribution depends on the image resolution, as the pixel width of the image limits the translational shifts used for their computation. To illustrate the effect of image pixel width, we use an experimental image of two co-localized signals shown in Fig. 2 *a*. For two co-localized signals, one expects a peak at the origin in the PCC profile; however, the computed profile does not show this behavior (Fig. 2 *b*). This discrepancy arises due to the dependence of image pixel width on the resolution and statistics of the joint product distributions that determine PDD and PCC profiles. The fact that the minimum separation distance can be either 0 or 1 pixel limits the resolution of these distributions. Further, due to low number of occupied pixels in the image, the product distribution suffers from low statistics. However, if the intensity of a single fluorescence signal covers multiple pixels (pixel width ≪ width of the signal or FWHM), one can interpolate the signal profile into smaller grids to improve both image resolution and statistics of the computed distributions. To achieve this, we divide each pixel area into smaller grids and interpolate the signal intensity using bi-linear interpolation as shown in Fig. 2 *c*. Fig. 2 *d* and *e* show PDD and PCC profiles for interpolated images with different pixel size. Interpolated images with lower pixel size lead to smooth PDD and PCC profiles. As a result, the PCC changes and recovers the profile of a co-localized signal (Fig. 2 *e*). The improvement in the PCC profile from image interpolation step saturates for smaller grid sizes, suggesting that further decrease of the pixel size does not improve the statistics of the distributions. This image enhancement step before image-based PCC computation is crucial to the interpret the experimental images and for comparison with the theoretical profiles derived in this work.

### PCC for multiple Gaussian signals

Next, we extend our analysis to multiple signals by considering two images, R and G, consisting of $N_{R}$ and $N_{G}$ Gaussian signals. The intensity, mean position, and standard deviation of a Gaussian signal is denoted by $p_{i}$, $\mu_{i}$, and $\sigma_{i}$ and $q_{j}$, $\mu_{j}$, and $\sigma_{j}$ for red and green signals, respectively. The images are defined as the sum of Gaussians as follows:

$$I_{R}\left(r\right)=\sum_{i}^{N_{R}}p_{i}\frac{1}{2\pi \sigma_{R}^{2}}\;\exp\;\left(-\frac{{\left(r-\mu_{i}\right)}^{2}}{2\sigma_{R}^{2}}\right)$$

and

(5) $$I_{G}\left(r\right)=\sum_{j}^{N_{G}}q_{j}\frac{1}{2\pi \sigma_{G}^{2}}\;\exp\;\left(-\frac{{\left(r-\mu_{j}\right)}^{2}}{2\sigma_{G}^{2}}\right)$$

where $p_{i}$ and $q_{i}$ are the amplitude of the individual signals in the image R and G respectively. Following the calculations for single pair distributions, one can write the PDD and PCC for multiple pairs as

(6) $$P\left(r\right)=\Delta r\frac{1}{\left(\sum_{i}p_{i}\sum_{j}q_{j}\right)}\sum_{i,j}p_{i}q_{j}\frac{r}{\sigma^{2}}\;\exp\;\left(-\frac{\mu_{i,j}^{2}+r^{2}}{2\sigma^{2}}\right)I_{0}\left(\frac{r\mu_{i,j}}{\sigma^{2}}\right)$$

where the sum is over all possible pairs (i.e., $N_{G}\times N_{R}$). Therefore, the PCC function is given by

(7) $$C\left(r\right)=\frac{A_{image}}{2\pi \sigma^{2}}\frac{1}{\left(\sum_{i}p_{i}\sum_{j}q_{j}\right)}\sum_{i,j}p_{i}q_{j}\;\exp\;\left(-\frac{\mu_{i,j}^{2}+r^{2}}{2\sigma^{2}}\right)I_{0}\left(\frac{r\mu_{i,j}}{\sigma^{2}}\right)\;\text{.}$$

**Figure 3:**
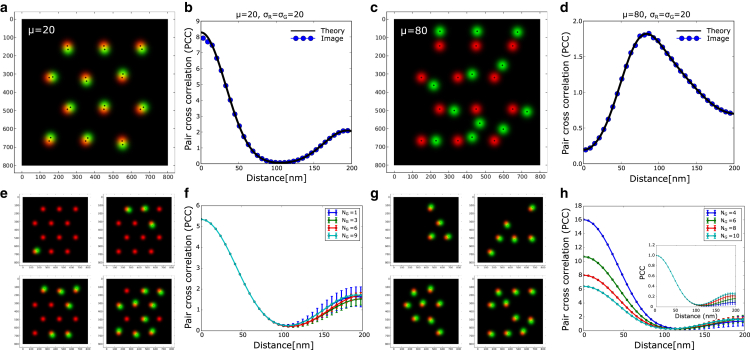
PCC analysis for multiple signal pairs. (*a*) Generated image with small separation (20nm) between red and green signals. (*b*) Comparison between analytical computed and image-based PCC profiles for correlated signal. (*c*) Generated image with a large separation distance (80nm) between red and green signals. (*d*) Comparison between analytical computed and image-based cross-correlation profiles for well-separated signals. (*e*) Generated image with different numbers of green molecules and fixed red molecules. (*f*) PCC is unchanged when the number density of one molecule is fixed. (*g*) Generated image with different number of pairs of red and green molecules. (*h*) PCC depends on the number of pairs. In the plot inset, the PCC is normalized by its maximum value for all the images.

The above expression matches the PCC computed from simulated images (generated using known $p_{i},q_{j},\mu$ and $\sigma =\sqrt{\sigma_{R}^{2}+\sigma_{G}^{2}}$ values) as shown in Fig. 3 *a–d*. To gain insight of the different terms, one can further simplify the PCC expression by neglecting contribution from the next-nearest neighbor of opposite color, i.e., $\mu_{ij_{next-nn}}\gg \sqrt{2\sigma }$ and assuming the separation distance with the first-nearest neighbor of opposite color to be similar, i.e., $\mu_{ij_{nn}}\approx \mu$. The second assumption holds when the signal pairs in the image have similar separation distances between them. We will discuss the case with heterogeneous separations later. Under these conditions, one obtains an expression that is a scaled version of single pair PCC and is defined by

(8) $$C\left(r\right)\approx \frac{A_{image}}{2\pi \sigma^{2}}\left(\frac{\sum_{i,j}p_{i}q_{i}}{\sum_{i}p_{i}\sum_{j}q_{j}}\right)\;\exp\;\left(-\frac{\mu^{2}+r^{2}}{2\sigma^{2}}\right)I_{0}\left(\frac{r\mu }{\sigma^{2}}\right)\text{.}$$

The expression leads to several insights into the dependence of PCC on the density of signal pairs. If $p_{i}\approx p,q_{j}\approx q$, i.e., the intensities of individual signals in the image are similar, then

(9) $$C\left(r\right)\approx \frac{A_{image}}{2\pi \sigma^{2}}\left(\frac{\min\left(N_{R},N_{G}\right)}{N_{R}N_{G}}\right)\;\exp\;\left(-\frac{\mu^{2}+r^{2}}{2\sigma^{2}}\right)I_{0}\left(\frac{r\mu }{\sigma^{2}}\right)\text{.}$$

Here, we have used $\sum_{i,j}p_{i}q_{j}=pq\;\min\left(N_{R},N_{G}\right)$, $\sum_{i}p_{i}=pN_{R}$ and $\sum_{i}q_{i}=qN_{G}$. The numerator of the scaling factor depends on the number of signal pairs, and the denominator depends on the product of the number of signals. If $N_{R}\neq N_{G}$, then $\min\left(N_{R},N_{G}\right)/\left(N_{R}N_{G}\right)=1/\max\left(N_{R},N_{G}\right)$. The expression implies that the PCC depends on the signal that is higher in number in the image and does not change if the number of another color signal decreases in the other image. We have illustrated this scenario with simulated images in Fig. 3 *e* and *f*, where, for a fixed number of red signals ($N_{R}=12$), the green signals are lowered in number ($N_{G}=1,3,6,9$). The profiles differ only for large distances where the contribution from second-nearest neighbor leads to some variation in the profile. Typically, a second peak in the full PCC arises due to the presence of next-nearest-neighbor signals of the opposite color (green/red) for a given color (red/green) if the signal pairs exhibit some regularity in their arrangement within the sampled image. The PCC profiles are the average of 10 simulated images where a fixed number green signals are generated at different random orientations. The variance in the PCC profile from multiple simulated images is shown in the plot. However, when the total number of signal pairs change, the PCC scales as 1/N (where $N_{R}=N_{G}=N$, as shown in Fig. 3 *g* and *h*), i.e., its value decreases, but its shape remains the same when the PCC profile is normalized with its maximum value (Fig. 3 *h* inset). This illustrates that the PCC profile remains largely independent of image area if the signals are homogeneously distributed, as an increase in sample area proportionally increases the number of pairs, balancing out their effects.

### Separation distance estimation in simulated images

The above results suggest that, for co-localizing signals, the value of PCC depends on multiple factors such as image size, signal number density, separation distance, and signal size. However the PCC profile shape depends mainly on the signal separation and signal standard deviation, i.e., the signal size. Therefore, to extract the average separation distance and signal size from the two-color images, one can fit the normalized PCC with the analytical expression in Eq. 8. The variation of PCC depends on distance *r* through two parameters *μ* and *σ*, which can be obtained from the fits. To accurately estimate these parameters, we perform conditional fitting by first estimating $\sigma_{R}$ and $\sigma_{G}$ from the individual images. We define self cross-correlation, where an image is cross-correlated with its copy, which mathematically means $\mu_{ij}=0$ and is given by

(10) $$C_{SS}\left(r\right)\approx \frac{A_{image}}{4\pi \sigma_{S}^{2}}\left(\frac{\sum_{i}^{N_{S}}p_{i}^{2}}{{\left(\sum_{i}^{N_{S}}p_{i}\right)}^{2}}\right)\;\exp\;\left(-\frac{r^{2}}{4\sigma_{S}^{2}}\right)$$

where S∈[R,G]. Therefore, one can estimate the effective *σ* for each signal by fitting the computed self cross-correlation function with the above function. Once both $\sigma_{R}$ and $\sigma_{G}$ are determined, the separation distance can be obtained by conditionally fitting the cross-correlations profile with the corresponding analytical function C(r) for single parameter *μ*.

**Figure 4:**
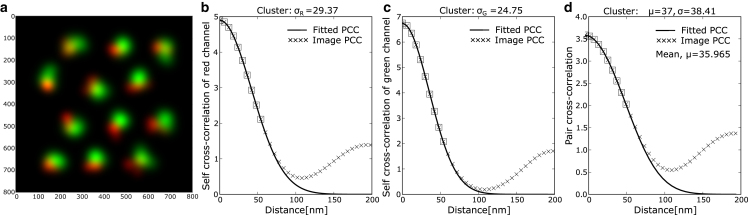
Fitting procedure for distance estimation. (*a*) Example image with small separation (30nm) between red and green signals. Here, we have simulated heterogeneously shaped signals using clusters of closely spaced localization points to generate individual signal spots. The resulting red and green signals are separated by a mean separation of μ = 36 nm. (*b*) The self-cross-correlation profile is computed by performing a PCC analysis of a signal with itself (i.e., PCC with zero separation distance). The profile depends only on the standard deviation of the signal. Fitting the measured profile with analytical expression, we estimate the standard deviation of red signal (estimated $\sigma_{R}=29.37\;nm$). (*c*) The steps in (*b*) are repeated for green signals to estimate the standard deviation (estimated $\sigma_{G}=24.75\;nm$). (*d*) Using the standard deviation of red and green signal, the standard deviation of the joint distribution is calculated. Then the measured PCC profile is fitted with the analytical expression of PCC with single parameter *μ*, the separation distance. The mean separation between the red and green signals is μ=35.965nm and the estimated separation distance of the cluster is 37nm.

To illustrate these steps, we have generated an image with multiple pairs of signals in Fig. 4. Furthermore, we have created images with heterogeneous signal shapes (larger than single-molecule signals) by creating clusters of focal red and green points. We generated the images by assigning red focal points on a hexagonal lattice and placing green focal points at a fixed distance away from them with random orientations. Then we added Gaussian signals at the focal points and a few points in their neighborhood to create red and green signals or clusters with different shapes (to account of signal heterogeneity in experiments). The mean separation between the red and green signals was measured. This is the separation distance between the centers of the effective red and green signals or clusters. We then applied our multi-step procedure to determine the mean separation by fitting the three profiles (two self-cross-correlations and one cross-correlation). Fig. 4 shows that the separation distance estimated from the fitting procedure is close to the mean separation. In the demonstrated simulated image, initial separation of between focal red and green signals is set to lattice separation of 30nm that increased to ≈36nm upon extending the signals to form cluster/large signals and the estimated separation from the fitting procedure came to be ≈37nm. Further, we observe that the image-based PCC (symbols in Fig. 4) from simulated images and the corresponding fitted analytical PCC (line) diverge at longer distances. The secondary peak in the PCC arises from the contribution of the next-nearest neighbors, which is considered negligible when deriving the analytical expression of PCC in Eqs. 8, 9, and 10. For this reason, we fit only the decreasing part of the PCC profile (open squares in all the PCC profile plots) with the analytical expression, as this region contains the dominant contribution from the first-nearest neighbor.

### Estimation of molecular separation in malaria-infected RBCs

**Figure 5:**
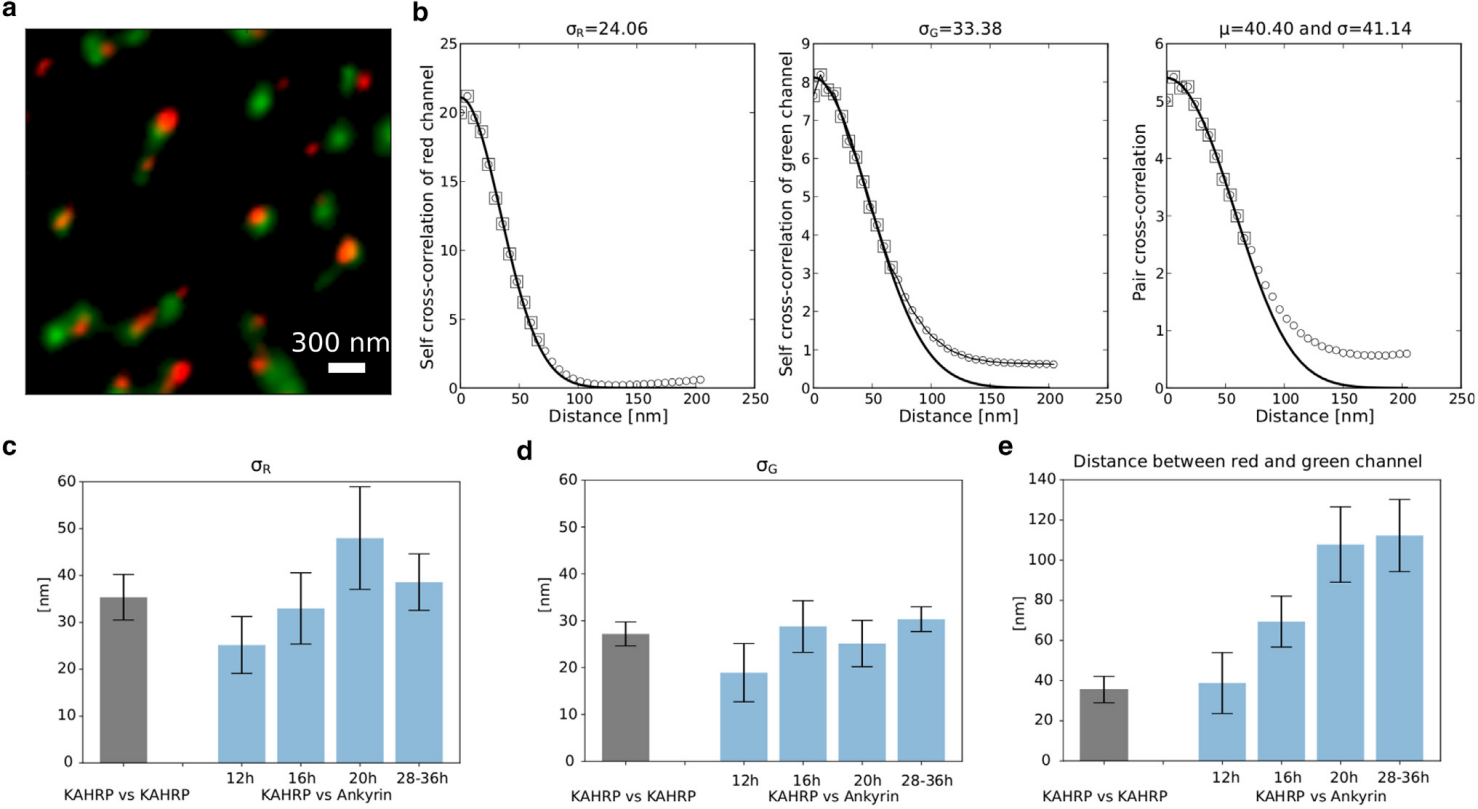
Extraction of separation distance by fitting experimental self-cross-correlation and cross-correlation profiles. (*a*) A representative STED images of exposed membranes prepared from *P. falciparum*-infected erythrocyte. The membrane is stained with two different antibodies against KAHRP (the rabbit peptide antibody pAb and the mouse monoclonal antibody mAb18.2). (*b*) Separation distance estimation involves three steps. First, the self-cross-correlation profiles of the two channels are fitted with the analytical function for self-cross-correlation to estimate the standard deviation of fluorophore signals. The left and middle panel shows fitting for red and green channels, respectively. Using the standard deviation of individual channels, a joint standard deviation is calculated. Then the analytical PCC function is fitted with the experimental profile with joint standard deviation to obtain the separation distance. (*c*) Plot showing the standard deviation of the red channel at a different times post malaria invasion. (*d*) The same is shown for the green channel. (*e*) Plot shows separation distance between KAHRPs (using two antibodies) and between KAHRP and ankyrin over the course of malaria infection. 20 images were used to generate bar graphs in (*c*) and (*d*). For KAHRP vs. ankyrin data, KAHRP and ankyrin are tagged with red and green antibodies, respectively. Representative STED microscopy images for different stages are provided in Fig. S2.

In this section, we apply our understanding and fitting procedure to estimate the separation of KAHRP and ankyrin during the malaria infection in RBC. Fig. 5 *a* shows a representative STED image of exposed membranes prepared from *P. falciparum*-infected erythrocyte. As a reference case, we consider an RBC-membrane stained with two different antibodies against KAHRP. Note that the two antibodies bind to different epitopes and thus the two signals are not identical even when binding to the same molecule. To estimate the separation distance between KAHRPs, we first fit the self cross-correlation profiles of the two channels with the analytical function for self cross-correlation. This gives estimates of the standard deviation of signals in each channel. The left and middle panels in Fig. 5 *b* show the fit for red and green channels, respectively. In the next step, we fit the analytical PCC function with the experimental profile with the effective standard deviation $\sigma =\sqrt{\sigma_{R}^{2}+\sigma_{G}^{2}}$ fixed. This fitting step provides the estimate for the separation distance *μ*. Fig. 5 *c* and *d* show the standard deviation of the red and green channels, respectively, at different times post malaria invasion for two cases (KAHRP vs. KAHRP and KAHRP vs. ankyrin at different hours post malaria infection). In contrast to our simulated images (as shown in Figs. 3 and 4), where a second peak in the PCC profile is observed due to the repetitive occurrence of next-nearest neighbors at fixed distances, the experimental images show a saturating trend (Fig. 5). This can be attributed to the well-known fact that signal pairs are heterogeneously distributed in infected RBCs, unlike the more regular arrangement observed in uninfected RBCs (11). Such a heterogeneous arrangement of next-nearest neighbors leads to contributions from multiple secondary peaks that smooth out when summed, resulting in either random correlation (PCC value close to 1, as in Fig. 5 *b*, middle panel, for green signals) or no correlation with the measured distance due to the large separation of the secondary signals (as in Fig. 5 *b*, left, for red signals).

**Figure 6:**
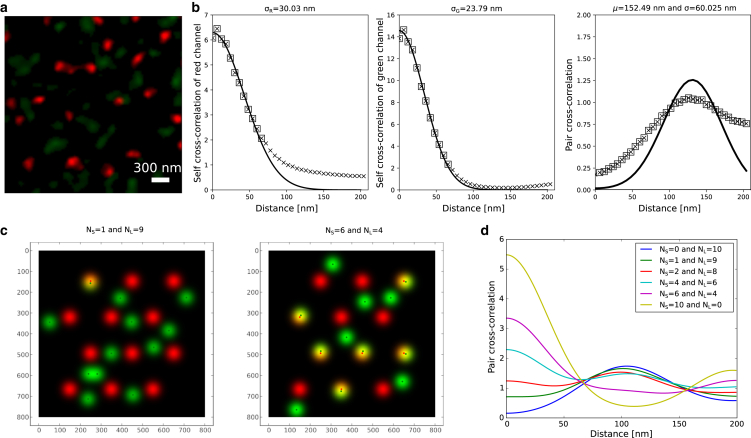
Effect of heterogeneous separation in the sample. (*a*) A representative STED images of exposed membranes prepared from *P. falciparum*-infected erythrocyte at 36h post invasion. The membrane is stained with KAHRP (the rabbit peptide antibody pAb; *red*) and ankyrin (mouse monoclonal ankyrin-1 antibody H-4; *green*). (*b*) The standard deviation for the red and green channels is estimated as before. The separation distance is evident from the experimental profile. However, the analytical profile does not fit well in this case. (*c*) Simulated data with heterogeneous separation distance between nearest neighbors. In the left image, the count of pairs with short (10nm) and large (100nm separation is 1 and 9, respectively. In the right image, the count of pairs with short and large separation is 6 and 4, respectively. (*d*) The change in the fraction of signal pairs with short and large separation dictates the pair correlation and leads to transition from peak at large separation to peak at the origin. This is illustrated in the plot with PCC values for different images with varying $N_{S}$ and $N_{L}$ values.

Using the described fitting procedure, we have estimated the separation distance between KAHRPs (using two antibodies) and between KAHRP and ankyrin over the course of malaria infection (Fig. 5 *e*). Our analysis shows that the separation distance between KAHRP and ankyrin clusters progressively increases during malaria infection, from 40nm (same as in the reference case of KAHRP vs. KAHRP) at the ring stage to over 100nm at the trophozoite stage. The smallest estimated separation of close to 40nm, seen in both KAHRP vs. KAHRP and KAHRP vs. ankyrin images, could be influenced of the optical setup (the spatial resolution of STED lies in the range between 35nm to 50nm) as well as by the size and orientation of the antibody complexes (primary and secondary antibodies, which are approximately 21nm). Further, we observe that the signal size of KAHRP (indicated by the changes in standard deviation in the red channel) increases more than 1.5 times than its values at the beginning of the infection. This finding agrees with previous studies suggesting that the parasite remodels the RBC membrane by increasing KAHRP clustering. The fit of experimental PCC with the analytic expression does not perfectly match the profile in the final stage (a representative image trophozoite stage shown in Fig. 6 *a* and *b*). This is because the separation distance between the molecule pairs becomes heterogeneous. However, in these conditions, the PCC has a clear peak that provides the estimation of separation distance between the signals.

To illustrate the effect of heterogeneous separation between signal pairs, we generated images with multiple signal pairs having either short (10nm) or large (100nm) separations (Fig. 6 *c*). The number of pairs with short and large separation is denoted by $N_{S}$ and $N_{L}$, respectively. The PCC profile shows a peak at a large distance (Fig. 6 *d* with $N_{S}=0$ and $N_{L}=10$). As the number of signal pairs with short distances is increased, the PCC profile broadens (Fig. 6 *d* with $N_{S}=1$ and $N_{L}=9$) and eventually shows a peak at origin as the number of signals with show distance is further increased. Therefore, we think the broadening of PCC profile in the experiments at large separation distance is due to variability in separation distance between the molecular pairs. The choice of short and large separation in the demonstrated case was made to ensure a clear distinction between the peaks at short separation (near the origin) and a well-separated secondary peak at large separation. For other combinations, where the two separation values are close to each other or the sample exhibits heterogeneous, multiple different separation values, it becomes challenging to disentangle these contributions from different pair separation values. In such samples, the measured separation distance from the fits can be thought of as the mean of all separations between pairs, but future work is required to better approach this experimentally important case.

## Discussion

With the recent advances in multi-modal imaging and super-resolution microscopy, it has become crucial to develop quantitative methods to analyze molecular-scale patterns within images and infer meaningful numbers. In this work, we provide a theoretical analysis of the image cross-correlation method that has been used in our previous study to quantify changes in molecular organization at RBC-membranes during malaria infection (10,12). Previous studies used cross-correlation method to estimate spatial co-localization of point localization-based imaging data. The PCC method was first applied to show spatial co-localization of glycosylphosphatidylinositol and actin in the presence of an antibody that cross-links them (8). In another study, PCC indicated that the co-distribution of a kinase and a peptide in CH27 mouse B cell lymphoma cell line changes in the absence and presence of an antigen (7). The cross-correlation method was also used to investigate spatial arrangement of several proteins in uninfected RBC membrane using two-color single-molecule localization from STORM imaging (11). All these studies defined cross-correlation on localization points to compare clustering and spatial co-localization and separation between two colors. Our previous work used the cross-correlation method on two-color STED images to compare changes in different labeled molecules over the course of malaria infection (10,12). However, all of these studies provide only qualitative analysis of molecular reorganization through changes in profiles of PCC function. To generalize the method to large datasets under different conditions, understanding the impact of image size, single-molecule density, and their signal shape is important. The present study makes progress in this direction by providing the theoretical underpinning of the PCC method of image analysis. To make analytical progress, we have used 2D Gaussian point-spread functions to simulate single-molecular signal and provide an analytical expression for PCC of a pair of two molecules. In principle, this approach could be easily extended to 3D, but it would be difficult to find analytical solutions for non-Gaussian point-spread functions.

Our analysis not only enhances the understanding of the PCC profiles but also allows us to estimate meaningful parameters from the analytical expression for PCC. First, we study the case of a single signal pair to understand how the presence or absence of a peak in the PCC profile, which qualitatively indicates co-localization, depends on signal parameters such as the size of individual molecules (i.e., standard deviation), the separation distance, and the image size. We find that this transition is continuous and depends solely on the separation distance and the sum of the variances of the two signals. We further extend this theoretical analysis to multiple signal pairs to quantitatively assess the effect of signal number density and their spatial distribution. This case provides insight into how the absolute value of the PCC is modulated in the presence of multiple signal pairs or their density. To validate our method, we apply this analysis to synthetically generated images with known parameters, exploring various possibilities for interpreting different types of image data that are usually not achievable in experiments. We found that the PCC profile is unchanged when the number density of one signal is decreasing, and the shape of the profile remains unchanged over a good range if the separation distance between signal pairs in the image is homogeneous, regardless of their number density.

The applicability of this methodology to image-based experimental data requires not only an analytical understanding but also an accurate determination of the PCC profile, which could be limited by image resolution. In our case, we find that image interpolation as a preprocessing step can improve the accuracy of the computed PCC profile from experimental images. We apply our theoretical analysis to quantify molecular changes in key proteins in RBC membrane images during malaria infection. Specifically, we observe that the co-localization of KAHRP and ankyrin diminishes and their separation increases as the malaria infection progresses. This change, previously reported qualitatively through PCC profiles, is now precisely quantified using our analysis method, revealing exact changes in molecular separation distance and average signal size. Specifically, we observe an increase in the signal size of KAHRP protein only during infection progression, which aligns with experimental literature suggesting increased clustering of KAHRPs to the actin junctions in the RBC cytoskeleton. These observations are critical as they suggest new underlying biological observation that might otherwise be overlooked through qualitative image analysis.

Our approach should be applicable to all systems with a certain degree of spatial regularity, which is typically the case for membrane-bound processes in locally flat cells, including the case of malaria-infected RBCs studied here. Because our aim is to extract statements on the nanoscale organization, it does not work for confocal datasets. In fact, we did examine confocal data on infected RBCs, which show, e.g., very good qualitative co-localization of the different labels for KAHRP, but it was not possible to extract meaningful statements on nanoscale separations, as it was to be expected given the optical resolution around 200 nm. However, our method should work for all super-resolution methods, including localization methods like STORM or PALM. In the supporting material, we discuss the mathematical equivalence between the image-based approach taken here and these localization-based approaches. It would be highly interesting to directly apply our method to both approaches using, e.g., the same sample imaged by different microscopy techniques. However, such a procedure is challenging to achieve with real biological samples. To demonstrate the potential of such an approach, we simulated STORM data from experimental images and compared the localization-based PCC profiles with the image-based PCC profiles for different numbers of single-molecule centroids in a given image. We found that the high density of point localization data provides accurate PCC profiles at all distances, consistent with those obtained from image-based PCC. The PCC profile deviates or lacks accuracy at short distances only when the density of localization points is low (Fig. S1). These results validate the utility of the method for estimating separation distances in a diverse set of images, where signal pairs have similar separations regardless of how the pairs are arranged within the image.

To make further progress in the quantitative analysis of two-color super-resolution imaging data, it would be interesting to use samples that allow precise positioning (e.g., using quantum dots or DNA origami) to further test the validity of our approach. For biological samples, it appears very important to also make progress with heterogeneous samples, as noted here for the case of late-stage infections, or, e.g., for protein co-localization in cells migrating through heterogeneous environments, which would also involve correlation integrals on curved surfaces. Because such challenges are hard to address with analytical approaches, machine learning might be a rewarding avenue for future work in this direction.

## Acknowledgments

We thank Mike Heilemann and Leon Lettermann for useful discussions. We acknowledge funding by the 10.13039/501100001659Deutsche Forschungsgemeinschaft (DFG, German Research Foundation) through Collaborative Research Center 1129 (Projektnummer 240245660).

## Author contributions

P.P. and U.S.S. designed the research. P.P. performed research and analyzed data. C.S. and M.L. designed and performed the experiments. U.S.S. and M.L. supervised the project. P.P. and U.S.S. wrote the original draft of the paper. All authors reviewed and approved the paper.

## Declaration of interests

The authors declare no competing interests.

## Supporting material

Document S1. Figures S1 and S2

Document S2. Article plus supporting material

## Footnotes

Supporting material can be found online at https://doi.org/10.1016/j.bpj.2025.03.002.
