# Supplementary material for: Pair cross-correlation analysis for assessing protein co-localization
Source: Biophys J. 2025 Mar 12;124(20):3396–407. doi: 10.1016/j.bpj.2025.03.002 (PMC12709269; doi:10.1016/j.bpj.2025.03.002)
Supplement: Document S1. Figures S1 and S2 [file mmc1.pdf]

Biophysical Journal, Volume *124*

Supplemental information

Pair cross-correlation analysis for assessing protein co-localization

Pintu Patra, Cecilia P. Sanchez, Michael Lanzer, and Ulrich S. Schwarz

Supporting Text : Pair cross-correlation analysis for assessing protein co-localization

Pintu Patra[1,2,3], Cecilia Sanchez[4], Michael Lanzer[4], and Ulrich S. Schwarz[1,2*]

[1]Institute for Theoretical Physics, Heidelberg University, Heidelberg, Germany
[2]BioQuant, Heidelberg University, Heidelberg, Germany
[3]Department of Physics, Indian Institute of Technology Kharagpur, Kharagpur, India
[4]Department of Infectious Diseases, Parasitology, Universitätsklinikum Heidelberg, Heidelberg, Germany
[*]schwarz@thphys.uni-heidelberg.de

PAIR CROSS-CORRELATION BETWEEN TWO IMAGES

Here we describe the computation of pair cross-correlation between two images. First, we define two-dimensional pair distance distribution $P(\rho)$ for given distance ρ as the normalized product of intensity values $I_R(x_i, y_j)$ of one image (red channel) with intensity values of another image at location $I_G(x_m, y_n)$ such that $\rho = \sqrt{(x_m - x_i)^2 + (y_n - y_j)^2}$. The normalization factor is the pixel-wise intensity product of the two images. Mathematically, $P(\rho)$ (1, 2) can be written as

$$P(\rho) = \frac{\sum_{i,j} \sum_{m,n} I_R(x_i, y_j) I_G(x_m, y_n) \delta\left(\sqrt{(x_m - x_i)^2 + (y_n - y_j)^2} - \rho\right)}{\sum_{i,j} \sum_{m,n} I_R(x_i, y_j) I_G(x_m, y_n)}. \tag{1}$$

For image dimensions $X \times Y$, the parameter ρ takes from 0 to $\sqrt{X^2 + Y^2}$ with intermediate values given by $\sqrt{(x_m - x_i)^2 + (y_n - y_j)^2}$ for $x_m - x_i = 0, 1, 2, ...X$ and $y_n - y_j = 0, 1, 2, ...Y$. Next, a histogram of pair distance distribution is computed by using bins of width Δr as

$$H(r, r + \Delta r) = \sum_{\rho=r}^{\rho=r+\Delta r} P(\rho). \tag{2}$$

The above distribution is further normalized to account for the increase in the area of radial bins. Specifically, the area of each bin with respect to the total image area, $N(r) = \pi \Delta r(2r + \Delta r)/A_{image}$ is used as a normalization factor (1, 3). This makes the distribution dimensionless and analogous to the cross-correlation function defined for localization points (3, 4). The resultant distribution is the cross-correlation distribution for two images,

$$C(r, r + \Delta r) = \frac{A_{image}}{\pi \Delta r(2r + \Delta r)} \sum_{\rho=r}^{\rho=r+\Delta r} \frac{\sum_{i,j} \sum_{m,n} I_R(x_i, y_j) I_G(x_m, y_n) \delta(\sqrt{(x_m - x_i)^2 + (y_n - y_j)^2} - \rho)}{\sum_{i,j} \sum_{m,n} I_R(x_i, y_j) I_G(x_m, y_n)}. \tag{3}$$

For all the experimental images analyzed in this work, we used image intensity thresholding (value = 20, with the maximum intensity value being 255) as the sole preprocessing step. In principle, PCC can be computed without the thresholding step, but we employed it primarily to accelerate the computation, which involves large matrix operations.
For localization-based data, the above expression for pair cross-correlation can be written in discrete form by representing the image as the sum of centroid locations of single-molecule fluorescence signals, using

$$I_R(r) = \sum_{i=1}^{n_R} \delta(\mathbf{r_i} - \mathbf{r}), \quad \mathbf{I_G}(\mathbf{r}) = \sum_{\mathbf{j}=1}^{\mathbf{n_G}} \delta(\mathbf{r_j} - \mathbf{r}). \tag{4}$$

The expression for PCC reduces to:

$$C(r, r + \Delta r) = \frac{A}{\pi \Delta r(2r + \Delta r)\ n_R\, n_G} \sum_{\rho=r}^{\rho=r+\Delta r} \sum_{i=1}^{n_R} \sum_{j=1}^{n_G} \delta(|\mathbf{r_i} - \mathbf{r_j}| - \rho). \tag{5}$$

Here, we have used the properties of delta functions. The summation term in the above expression is computed by finding the histogram of pairwise distances of single-molecule localization points from two channels (1, 3–5). The average pairwise distance distribution for a randomly distributed dataset is given by $\pi \Delta r(2r + \Delta r) n_R n_G / A$(1, 3). Thus, the pair cross-correlation function approaches 1 for such datasets.

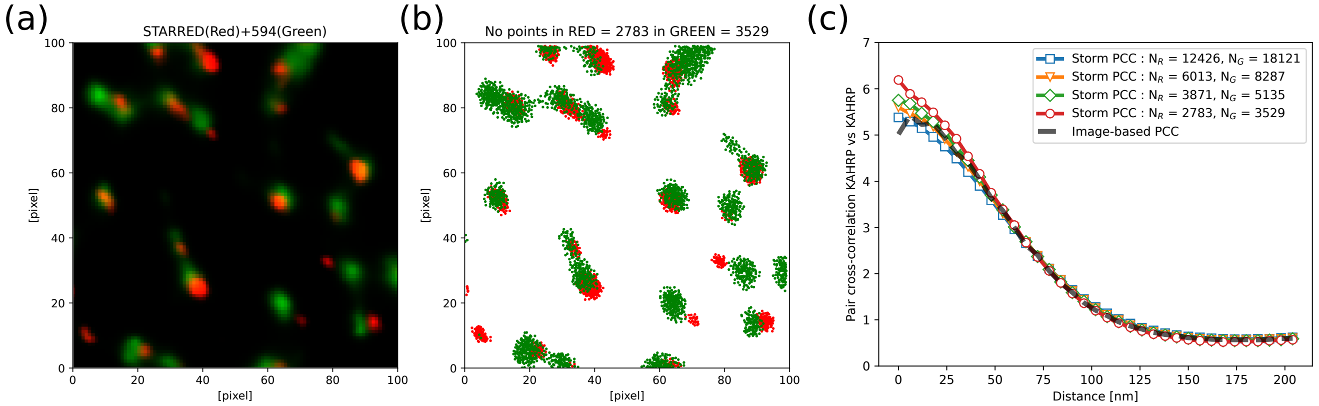

Fig. S1: (a) STED microscopy image of two different antibodies against KAHRP (same as in Fig. 5 a). 1 pixel equals to 15 nm. (b) STORM image data containing red and green localization points generated using the STED image as a probability map. The number of red and green points for the two channels is indicated in the title. (c) Comparison of the image-based PCC of the STED image (dashed line) with the PCC computed from the generated STORM data for different numbers of localization points (line and points).

Generation of simulated STORM data

To generate simulated STORM data from experimental images, we assign localization points to each pixel (randomly within the pixel) in proportion to its intensity. The proportionality factor determines the total number of points assigned. To distribute the calculated number of points within a given pixel, we use intensity interpolation to stochastically assign points within the pixel. Fig. S1 shows the comparison of the image-based PCC (dashed line) with the PCC computed from the generated STORM data for different numbers of localization points (line and points).We find that the PCC values from both methods match when there is a large number of localization points. The correspondence is lower in cases with fewer localization points for the two channels.

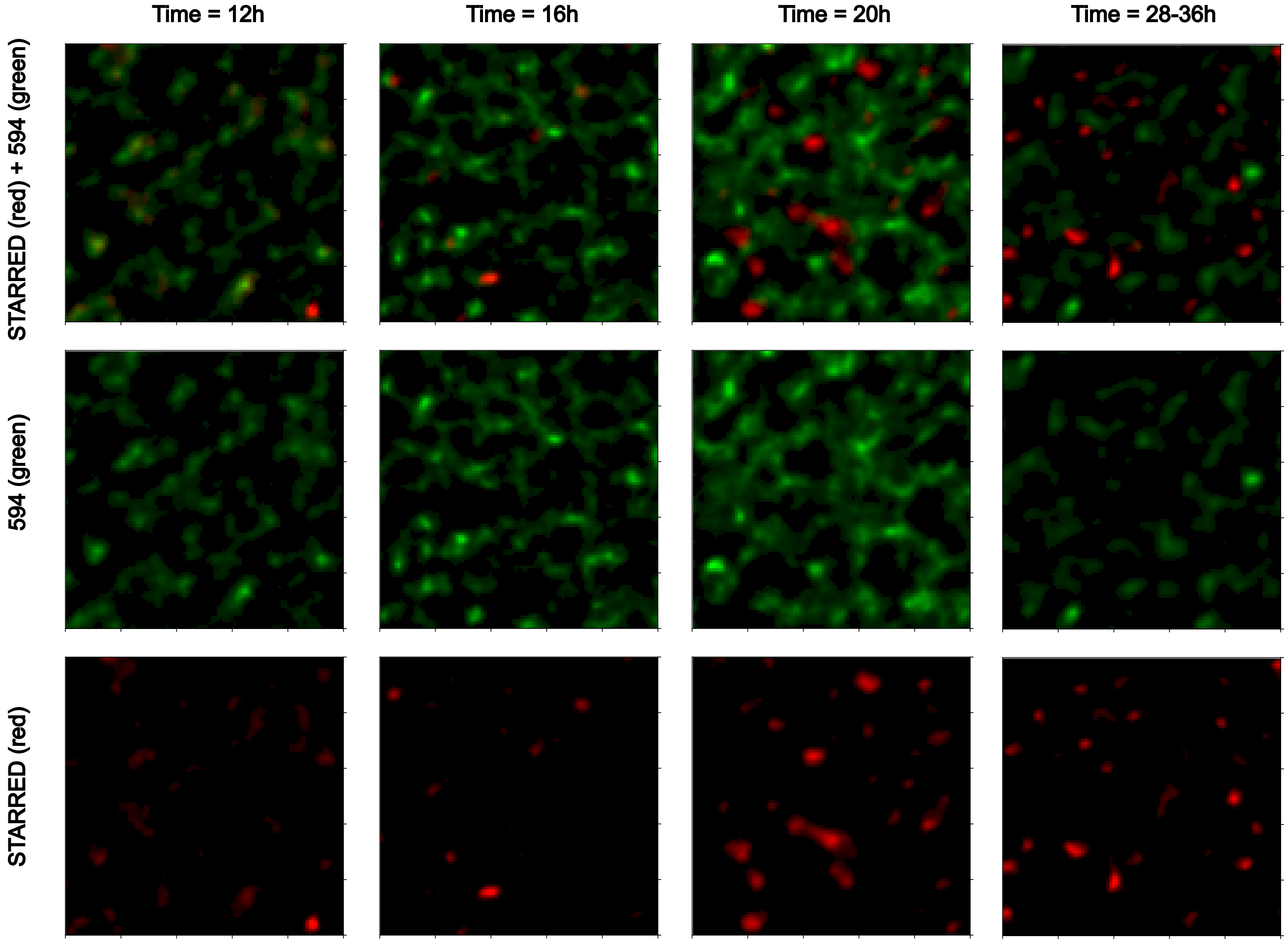

Fig. S2: Representative STED microscopy images of KAHRP (red) and ankyrin (green) at different times over the course of malaria infection. 20 images were used to generate bar graphs in Fig. 5 c-d.

REFERENCES

1. Schnitzbauer, J., Y. Wang, S. Zhao, M. Bakalar, T. Nuwal, B. Chen, and B. Huang, 2018. Correlation analysis framework for localization-based superresolution microscopy. *Proceedings of the National Academy of Sciences* 115:3219–3224.

2. Churchman, L. S., Z. Ökten, R. S. Rock, J. F. Dawson, and J. A. Spudich, 2005. Single molecule high-resolution colocalization of Cy3 and Cy5 attached to macromolecules measures intramolecular distances through time. *Proceedings of the National Academy of Sciences* 102:1419–1423.

3. Stone, M. B., and S. L. Veatch, 2015. Steady-state cross-correlations for live two-colour super-resolution localization data sets. *Nature communications* 6:7347.

4. Pan, L., R. Yan, W. Li, and K. Xu, 2018. Super-resolution microscopy reveals the native ultrastructure of the erythrocyte cytoskeleton. *Cell reports* 22:1151–1158.

5. Sengupta, P., T. Jovanovic-Talisman, D. Skoko, M. Renz, S. L. Veatch, and J. Lippincott-Schwartz, 2011. Probing protein heterogeneity in the plasma membrane using PALM and pair correlation analysis. *Nature methods* 8:969.
